# Comparative Analysis of Coronal Sealing Materials in Endodontics: Exploring Non-Eugenol Zinc Oxide-Based versus Glass-Ionomer Cement Systems

**DOI:** 10.1055/s-0044-1782695

**Published:** 2024-06-28

**Authors:** Mohamed Hashim Alamin, Sara Ayman Yaghi, Abdullah Faris Al-Safi, Wared R. Y. R. Bouresly, Kausar Sadia Fakhruddin, Lakshman Perera Samaranayake, Saaid Al Shehadat

**Affiliations:** 1 Department of Restorative Dentistry, College of Dental Medicine, University of Sharjah, Sharjah, United Arab Emirates; 2 Department of Orthodontics, Pediatric and Community Dentistry, College of Dental Medicine, University of Sharjah, Sharjah, United Arab Emirates; 3 Department of oral biosciences, University of Hong Kong, Hong Kong; 4 Department of Periodontology, Chulalongkorn university, Bangkok, Thailand

**Keywords:** temporary coronal sealers, sealing properties, microleakage, glass-ionomer cement, zinc oxide-based materials, root canal treatment

## Abstract

The proper closure of the access cavity between appointments during endodontic treatment is paramount and relies on temporary fillings. This systematic review evaluates the effectiveness of zinc oxide-based materials and glass-ionomer cement (GIC) as temporary coronal sealers after root canal treatment in extracted human teeth. Three databases were searched to identify randomized clinical trials that examined the sealing properties of various temporary sealing materials using dyes or stains as indicators. A total of seven *in vitro* studies that fulfilled the eligibility criteria were critically analyzed. These indicated significant variations in the relative sealing ability of the coronal breach of endodontically treated teeth, either by zinc oxide or GIC-based materials. While GIC-based material (e.g., Fuji IX and Fuji II) exhibited superior sealing of single-rooted teeth, zinc oxide-based material (e.g., Cavit, Coltosol, Caviton) also showed promising attributes. Resin-modified GIC formulations displayed enhanced physical properties, yet challenges related to adhesive failure and shrinkage during polymerization were observed. Zinc oxide-based materials have demonstrated superior coronal sealing effectiveness over certain GIC in controlled settings. Their premixed nature ensures consistent application and hygroscopic properties improve cavity sealing. However, the focus on dye penetration tests for microleakage *in vitro* may not fully represent the risk of bacterial infiltration. Thus, *in vivo* studies are crucial for validating these findings in clinical contexts.

## Introduction

The success of endodontic treatment relies on the effective removal of bacteria and pulp tissue through chemical and mechanical debridement, as well as the establishment of a hermetic seal during root canal obturation. 1 Coronal microbial infiltration subsequent to an endodontic intervention can potentially contribute to the long-term failure of treatment and the development of apical periodontitis, even in the presence of a radiographically satisfactory root filling. 2 Therefore, it is crucial to employ temporary coronal sealers to prevent contamination of the root canal system during the period between endodontic and restorative appointments. These coronal sealers serve the purpose of impeding the ingress of contaminant bacteria, and moisture, safeguarding the root canal system. In multivisit treatments, the proper closure of the access cavity between appointments using a temporary filling plays a critical role in the treatment outcome. 3 The temporary endodontic filling material must meet specific requirements, such as preventing bacterial contamination of the pulp space and leakage of root canal dressing or saliva. The primary objective of an endodontic temporary filling material is to maintain a tight seal at the coronal junction of the tooth and the oral milieu during the entire endodontic treatment process. 4

The ideal sealing material should exhibit desirable characteristics to ensure its effectiveness. These include minimal solubility, postplacement dimensional stability, and antibacterial properties. 5 Additionally, it should possess optical visibility, allowing for easy identification of margins during removal without causing damage to the tooth structure or interim restorative material. 4 Currently, there are two major options available; non-eugenol zinc oxide-based materials such as zinc oxide-calcium sulfate material (Cavit) and a zinc oxide zinc sulfate-based material called Coltosol. Another option is glass-ionomer cement (GIC) which is a bioactive and adhesive dental materials option used as a root canal sealer.

Zinc oxide-based materials come in premixed forms, which provide a convenient option for chairside application and easy manipulation within the access cavity. 6 GIC, on the other hand, has the advantage of establishing a bond with dentin. This bond not only enhances its retention but also enables the controlled release of fluoride over time. This controlled fluoride release contributes to the prevention of secondary caries formation while maintaining the strength and biocompatibility of the material. Additionally, GICs exhibit antibacterial properties against a spectrum of bacterial species. 7

A perusal of the literature indicated that no recent reviews are available comparing the relative efficacy of zinc oxide-based and GIC-based materials as an effective root canal sealing material. Hence, the primary objective of this study was to conduct a systematic review of the existing literature on the transient coronal sealing efficacy of non-eugenol zinc oxide-based materials and glass-ionomer materials, utilized for the interim coronal sealing of teeth during endodontic treatment.

## Methods

### Data Sources

A systematic review was conducted using PubMed, ProQuest One Academic, and EBSCO host databases for the English language article to identify randomized clinical trials (RCTs) and *in vitro* and *ex vivo* studies from peer-reviewed journals.

### Search Terms

The English search terms used to select qualifying studies from the database published between January 1, 2000 and January 31, 2023, are as follows:

**#** 1 (temporary coronal sealing [MeSH) OR temporary dental fillings OR coronal sealants OR restorative materials OR interim restorations AND root canal treatment OR multi-visit endodontics OR endodontic-treated tooth AND coronal microleakage OR marginal leakage OR microleakage assessment OR sealing ability).
**#** 2 (zinc oxide-based [MeSH] OR non-eugenol-zinc oxide OR Zinc oxide eugenol alternatives OR non-eugenol- zinc oxide-based zinc sulfate OR zinc oxide-calcium sulfate OR multi-visit endodontics OR endodontic-treated tooth AND coronal microleakage OR marginal leakage OR sealing ability OR temporary dental fillings OR coronal sealants).
**#** 3 (glass ionomer cement OR GIC [MeSH) OR conventional glass ionomer cement OR resin-modified glass ionomer cement OR RMGIC OR high-viscosity Glass Ionomer Cement OR reinforced glass ionomer cement OR Giomer OR temporary coronal sealing OR restorative materials OR interim restorations OR compomer OR AND root canal treatment OR multi-visit endodontics OR endodontic-treated tooth AND coronal microleakage OR marginal leakage OR sealing ability OR temporary dental fillings OR coronal sealants).

Finally, the studies were selected based on the combination of the following search terms: # 2 # 1 AND # 2 AND # 3 AND #1 AND #3.

## Summary Measure

The main objective of this research study was to conduct a systematic review of the available data regarding the coronal sealing efficiency of different zinc oxide-based materials and GIC in the context of temporary coronal sealing of endodontically treated teeth.

### Study Selection

The following criteria were applied for the selection of studies:

(1) Inclusion criteria:
  - Studies that compared non-eugenol zinc oxide-based materials with various GIC materials for temporary coronal sealing of endodontically treated teeth.
  - Studies conducted on human extracted teeth reporting on microleakage assessment.
  - RCTs focusing on temporary coronal sealers.
  - English language articles.
  - Studies that assessed *in vitro* coronal seal leakage or microbial ingression in the temporary coronal seal.
(2) Exclusion criteria
  - Nonrandomized studies.
  - Studies not conducted on human teeth.
  - Review articles and case reports lacking comprehensive outcome details.
  - Studies not reporting on microleakage assessment.
  - Studies that involved eugenol-zinc oxide sealing materials.
  - Studies that did not align with the predetermined study objectives or consisted of abstracts only.
  - Non-English literature.

### Electronic Data Search and Analysis

**Fig. 1:**
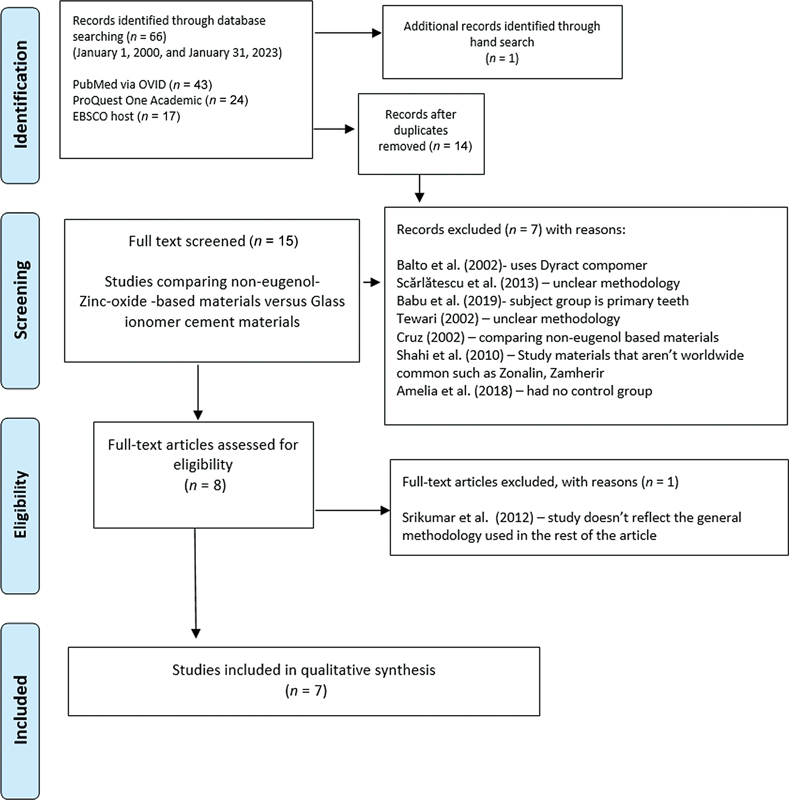
Preferred Reporting Items for Systematic Reviews and Meta-Analyses (PRISMA) flowchart of the literature search and study selection.

To ensure a systematic and comprehensive approach, we adhered to the Preferred Reporting Items for Systematic Reviews and Meta-Analyses guidelines. 8 9 The search strategy implemented and the outcomes obtained are illustrated in Fig. 1 .

During the initial stage of our three-step electronic data search and analysis, the titles and abstracts of relevant studies were assessed to identify those that met the predefined inclusion criteria. In the second stage, a detailed examination of the full texts of the identified articles was conducted to obtain a comprehensive understanding of the data. This rigorous analysis ensured that the selected studies met the eligibility criteria and reported outcomes aligned with the objectives. Additionally, a backward search was performed by scrutinizing the references of the included studies to identify any other relevant research.

In the final stage, the reviewers (S.Y. and W.B.) extracted and evaluated the data from the selected studies. Specific characteristics of each included study, such as study setting, samples, intervention, and country, were recorded using the Cochrane model. Other factors such as sample size, evaluation time, assessment methods, and study conclusions were systematically examined to synthesize the finding effectively.

To manage the identified manuscripts, the researchers employed bibliographic software called Endnote version 9 (Clarivate Analytics, United States). Table 1 summarizes the characteristics of the included *in vitro* trials and the reported results regarding the coronal sealing efficacy of the zinc oxide-based and GIC materials. This table offers a concise overview of the key findings from each study, facilitating the data analysis.

**Table 1:** Characteristics of the included studies and results

Study year Study design	Setting (no. of teeth)	Test and control groups	Inclusion and exclusion criteria	Test material	Type of assessment (dye penetration) (microbiological assessment)	Time points	Summary intervention (high/low)	Summary comparator (high/low)	Outcome
Balto (2002) *In vitro* 3	38	Teeth were randomly assigned into groups with test materials—moreover, there were two positive and negative controls, respectively, ( *n* = 4)	X	Zinc oxide-zinc sulfate-based (Cavit) versus GIC (Dyract)	Dye penetration Microleakage (none) Microbiological assessment ( *S. faecalis* and *C. albicans* )	4 wk	Cavit showed a low mean (±SD) value of 16.4 (±1.34). *S. faecalis* used as a microbial tracer	Dyract showed a higher mean (±SD) value of 17 (±4.9) compared to Cavit	No significant difference was observed in sealing abilities between the tested materials
Madarati et al (2008) *In vitro* 11 a	135	Teeth were randomly assigned into groups with test materials. There were ( *n* = 10) positive controls in the group	X	Zinc oxide-calcium sulfate-based (Coltosol) versus GIC (chemical cured)	Dye penetration Microleakage (mm) Microbiological assessment (none)	4 wk	Coltosol showed a high mean (±SD) value in the first week 1.28 (±0.33) and low value in the third week 2.30 (±0.90) compared to GIC	GIC showed a low mean (±SD) value in the first week 0.76 (±0.56) and high value in the third week 3.29 (±2.11) compared to Coltosol	Both the tested materials demonstrated some degree of marginal leakage. Coltosol performed better over time compared to GIC
Ciftçi et al (2009) *In vitro* 13 a	48	Teeth were randomly assigned into groups with test materials. With two positive and negative controls, respectively	X	Zinc oxide–calcium sulfate-based (Cavit-G) versus GIC (Ketac Molar Easymix)	Dye penetration Microleakage (score) Microbiological assessment (none)	1 wk	Cavit-G showed a low penetration score and a better seal	GIC specimens showed a high penetration score representing more marginal leakage	Both the tested materials demonstrated some degree of marginal leakage. However, Cavit-G showed less marginal leakage than GIC
Pieper et al (2009) *In vitro* 16 a	40	Teeth were randomly assigned into groups with test materials	X	Zinc oxide-zinc sulfate-based (Cavit) versus GIC (Vidrion R)	Dye penetration Microleakage (score) Microbiological assessment (none)	1 wk	Cavit showed a low score representing a better marginal seal	Vidrion R showed a comparatively high score representing a low marginal seal	Both tested materials showed different degrees of marginal leakage
Križnar et al (2016) *In vitro* 14	128	Teeth were randomly assigned into groups with test materials. With two positive and negative controls, respectively	X	Zinc oxide-zinc sulfate-based (Cavit) versus GIC (Fuji II LC, Fuji IX)	Dye penetration Microleakage (none) Microbiological assessment ( *S. mutans* culture leaked sample %)	90 d	Cavit showed the lowest bacterial penetration and the better seal (33.3%)	The Fuji II LC (66.7%) and Fuji IX (88.2%) test material showed the highest bacterial penetration values demonstrating weaker seals	Cavit demonstrated a better seal than Fuji II, and Fuji II had a better seal than Fuji IX
Milani et al (2017) *In vitro* 15 a	72	Teeth were randomly assigned into groups with test materials	X	Zinc oxide-calcium sulfate-based (Coltosol) versus GIC –Compoglass	Dye penetration Microleakage (score) Microbiological assessment (none)	2 mo	Coltosol showed a lower mean value (±SD), which is 1.5 (±0.53) in the first week, 2.12 (±0.354) in the first month, and 2.37 (±0.052) at the 60 days' evaluation time point	Compoglass showed a high mean value (±SD) which is 2.62 (±0.51) in the first week, 2.75 (±0.463) in the first month, and 2.87 (0.354) at 60 days' evaluation time point	Coltosol provided a more favorable seal compared to Compoglass
Hajaj et al (2021) *In vitro* 12	42	Teeth were randomly assigned into groups with test materials, with ( *n* = 2) teeth in the control group	X	Zinc oxide-calcium sulfate-based (Coltosol) versus GIC and light-cured compomer mGIC	Dye penetration Mean absorbance value Microbiological assessment (none)	2 wk	Coltosol F showed a mean value (±SD) 0.025 (±0.001) showing a weaker seal	GIC showed a low mean value (±SD) of 0.016 (±0.001). Light-cured compomer mGIC had a mean value of 0.014 (±0.001), showing a better seal	No significant difference was observed in sealing abilities between the tested materials. mGIC had a lower microleakage score compared to GIC

Abbreviations: GIC, glass-ionomer cement; SD, standard deviation.

Note: X = yes.

a Presence of thermocycling.

## Quality and the Overall Risk of Bias Assessment of the Included Reports

The quality and overall risk of bias assessment of the included reports were conducted independently by two assessors (K.S.F. and S.S.). In case of any discrepancies, the third and fourth reviewers (S.S. and K.S.F.) were consulted to reach a consensus. The hierarchy of evidence was followed, and *in vitro* laboratory trials were considered to provide the weakest level of evidence. This is because such trials may have limitations such as potential “false-positive” outcomes, limited external applicability, and inadequate generalization to clinical scenarios. 10

To evaluate the transparency and quality of the laboratory trials included in the systematic review, the researchers used the Consolidated Standards of Reporting Trials (CONSORT) guidelines. Accordingly, various aspects such as sample size, specimen preparation and handling, allocation sequence, randomization, and blinding were meticulously evaluated to determine their eligibility for inclusion in the systematic review ( Table 2 ).

**Table 2:** Risk of bias of the included studies

Study	Selection bias Baseline characteristics similarity/appropriate control selection	Selection bias Allocation concealment	Selection bias Randomization	Performance bias Blinding of researchers	Detection bias Blinding of outcome assessors	Reporting bias Selective outcome reporting	Incomplete outcome data (attrition bias)
Balto (2002) 3	**+**	**+**	**?**	**?**	**+**	**?**	**+**
Madarati et al (2008) 11	**+**	**?**	**?**	**?**	**?**	**+**	**+**
Ciftçi et al (2009) 13	**+**	**?**	**?**	**?**	**?**	**+**	**+**
Pieper et al (2009)	**+**	**+**	**+**	**+**	**+**	**+**	**+**
Križnar et al (2016) 14	**+**	**?**	**?**	**?**	**?**	**+**	**+**
Milani et al (2017) 15	**+**	**?**	**?**	**?**	**?**	**+**	**+**
Hajaj et al (2021) 12	**+**	**?**	**?**	**?**	**?**	**+**	**+**

Note: Risk of bias legends: + (low risk); – (high risk); ? (unclear risk).

## Results

A total of seven *in vitro* studies 3 11 12 13 14 15 16 were deemed eligible for the current systematic review. The included trials encompassed a total of 378 extracted human teeth. All included studies described the coronal sealing ability of zinc oxide-based materials and GIC used as temporary restorations in extracted human teeth. The assessment of seal quality in different temporary sealing materials has been conducted in these studies using stains or dyes. Only one study has employed microbiological evaluation to evaluate leakage and contamination. 14 The period of microleakage assessment duration ranged from 1 week to 3 months in the reviewed studies. The composition and characteristics of the included studies and the reported outcomes of the temporary coronal sealing material are summarized in Tables 1 and 3 .

**Table 3:** The composition of the tested materials

Material	Composition	Manufacturer	Studies
Zinc oxide-based material	Coltosol (Zinc oxide and zinc sulfate based)	(Coltosol, Colten, Langenau, Germany) (Coltene, Altstatten, Switzerland)	Madarati et al (2008) 11 Milani et al (2017) 15 Hajaj et al (2021) 12
	Cavit (Zinc oxide and calcium sulfate-zinc sulfate-based cement	(3M ESPE, Germany)	Križnar et al (2016) 14 Pieper et al (2009) 16 Balto (2002) 3
	Cavit G (Zinc oxide and calcium sulfate based)	(3M ESPE, Germany)	Ciftçi et al (2009) 13
Glass-ionomer cement	Dyract (Strontium alumino-fluorosilicate glass and resin matrix)	(Dentsply-De Trey, Konstanz, Germany)	Balto (2002) 3
	Compoglass (Glass ionomers and composites with polymer matrix and fillers)	(Vivadent, USA)	Milani et al (2017) 15
	Vidrion R ( *Powder* : aluminum-silicate glass *Liquid* : copolymers of polyacrylic acid, itaconic acid and tartaric acids)	(SS White, India)	Pieper et al (2009) 16
	GIC ( *Powder* : fluoroaluminosilicate glass *Liquid* : polyacrylic acid)	(Carefil-PL, Dentcare, Romsey, UK) (Kavitan Plus, PENTRON, Orange, USA)	Madarati et al (2008) 11 Hajaj et al 2021
	Light cured compomer mGIC (fluoroaluminosilicate glass and polyacrylic acid)	(DMG, Hamburg, Germany)	Hajaj et al (2021) 12
	Ketac Molar Easymix Fluorosilicate glass and polialquenoic and tartaric acid)	(3M Espe, Seefeld, Germany)	Ciftçi et al (2009) 13
	Resin-modified GIC Fuji II (fluoroaluminosilicate glass and polyacrylic acid)	(GC EUROPE, Belgium Leuven)	Križnar et al (2016) 14
	Conventional GIC Fuji IX (fluoroaluminosilicate glass and polyacrylic acid)	(GC EUROPE, Belgium Leuven)	Križnar et al (2016) 14

Abbreviation: GIC, glass-ionomer cement.

The materials evaluated in all the studies comprised zinc oxides, zinc sulfate-based compounds, self-cure temporary materials such as Cavit, Cavit G, and Coltosol, and GIC types, Compoglass (a combination of glass ionomers and composites with polymer matrix and fillers), conventional GIC Fuji IX, resin-modified GIC (RMGIC) Fuji II, and GIC formulations (consisting of fine-grained glass silicate ionomer and polyacrylic acid).

Three of the reviewed studies compared the sealing ability of GIC-based material and zinc oxides, and zinc sulfate-based compounds (Coltosol). In one study, the researchers evaluated the microleakage of GIC and Coltosol, at three different time intervals of 1, 2, and 4 weeks. 11 The results showed that GIC and Coltosol had the lowest mean microleakage values after 1 week, measuring 0.75 and 1.28 mm, respectively. However, the sealing ability of both materials noticeably declined over the 4-week observation period.

In contrast, Milani et al 15 compared the sealing ability of Coltosol and Compoglass (a compomer) over 1 week, 1 month, and 2 months. They found that after 2 months, there were no significant differences in the sealing ability of the tested materials. However, at the 1-week and 1-month intervals, Coltosol exhibited superior sealing performance compared to Compoglass.

Hajaj et al 12 compared the characteristic sealing in single-rooted and multirooted teeth and found that GIC exhibited superior sealing performance in single-rooted teeth compared to Coltosol.

Križnar et al 14 conducted a comparative study to assess the sealing capabilities of Cavit, Fuji II (RMGIC), and Fuji IX (conventional GIC). The findings indicated that Cavit demonstrated the longest duration of coronal seal, which was approximately 70 days. In contrast, Fuji II exhibited a sealing duration of 43 days before experiencing leakage, while Fuji IX had the shortest duration of only 21 days. Interestingly, none of the materials subjected to scrutiny achieved complete prevention of bacterial microleakage.

Furthermore, in another previous *in vitro* investigation, Balto in 2002, 3 investigated the efficacy of Cavit and Dyract (a light-cured compomer) in sealing the coronal breach. This assessment involved the utilization of microbial tracers, namely, *Streptococcus faecalis* and *Candida albicans* . The findings revealed similar outcomes in terms of microbial penetration for either a zinc oxide or a zinc sulfate-based compound, and a compomer.

In a separate study conducted by Ciftçi et al, 13 a comparative appraisal was conducted to assess the efficacy of two different materials, namely, Cavit-G (a zinc oxide-calcium sulfate-based material) and CIS (an enhanced GIC with a higher powder-to-liquid ratio). The outcome of this study indicated that the former compound characterized by its composite of zinc oxide and zinc sulfate-based compounds exhibited significantly lower scores for marginal integrity of the seal compared to the CIS groups.

However, in another study by Pieper et al, 16 focusing on the marginal seal, it was reported that Cavit demonstrated superior performance to Vidrion R, a GIC filling material. Notably, both Cavit and the GIC exhibited a distinct wear pattern of abrasive wear, characterized by a substantial mass loss following brushing. Moreover, it merits mention that Cavit displayed significantly higher levels of water sorption and solubility when compared to GIC.

## Discussion

The review encompassed different brands of zinc oxide-based materials and GIC as temporary coronal sealers in endodontic treatment ( Table 1 ). Our findings are of practical significance for clinicians engaged in multivisit endodontic procedures, as selecting an appropriate temporary restoration material plays a critical role in the success of endodontic therapy. To the best of our knowledge, this review represents the first attempt to assess the effectiveness of various zinc oxide-based materials in coronal sealing compared to GIC, specifically in the context of their application as temporary restorations after root canal treatment.

These *in vitro* investigations have shed light on the varying outcomes associated with the relative effectiveness of GICs and zinc oxide-based materials as coronal sealing agents.

Almost all of the evaluated studies employed colored stains or dyes to assess the seal quality of various temporary sealing materials. Among the five studies examined, only Hajaj et al 12 investigated the sealing properties of GIC in both single-rooted and multirooted teeth. Their findings revealed superior sealing performance in single-rooted teeth compared to zinc oxide-based materials, specifically Coltosol.

GICs have attracted clinical interest due to their adhesive and bioactive properties, as well as some therapeutic effects. Their ability to bond to dentin, release fluoride without compromising material strength, 17 and biocompatibility make them advantageous for both restorative dentistry and endodontic procedures.

Following root canal treatment, GIC can be applied for the coronal sealing of multirooted teeth. 18 However, the reviewed study 12 has not extensively addressed crucial factors, including challenging tooth anatomy, load-bearing requirements, adaptability, and long-term durability. Additional research is required to gather conclusive evidence regarding the effectiveness of either of the tested material types in achieving successful sealing in multirooted teeth.

Highly viscous formulations, such as Fuji IX and Fuji II, are examples of RMGICs, which are modifications of conventional GICs. RMGICs were developed to enhance the physical properties to mimic those of resin composites and resin cements while retaining the fundamental characteristics of conventional GICs. 19 These materials undergo polymerization and acid-base reactions during setting, combining the benefits of both conventional GIC and resin-based materials. 19

In comparison to RMGICs, Cavit, a hygroscopic material based on zinc oxide and calcium sulfate, exhibits a high linear expansion coefficient due to its water absorption properties. This characteristic is deliberately incorporated into its formulation to facilitate its setting reaction and provide a firm marginal seal, 6 making it well-suited for the purpose.

Though the sealing of the tooth-restoration interface is attributed to the expansion resulting from water diffusion, however, this also facilitates the swelling of components within water-filled spaces. 20 As Pieper et al's study in this review reveals, the significantly higher water sorption and solubility attributes in Cavit relative to GIC material explains the higher solubility observed in Cavit.

Križnar et al 14 reported that Cavit demonstrated the longest seal duration of 90 days compared to GIC-based material, specifically Fuji II (RMGIC) type. In addition, the *in vitro* bacteriological model study revealed that conventional GIC Fuji IX exhibited the least resistance to bacterial leakage, followed by RMGIC. 21 The outcomes inferred from the microbiological evaluation utilized in the mentioned study hold greater relevance for extrapolating the findings to clinical contexts. 21 It is disappointing to note that only the latter group of workers have used microbiological assessment in coronal leakage studies related to endodontics, and we believe that further work is essential to corroborate the available sparse data set on this important clinical feature.

The suboptimal performance of RMGIC materials can be attributed to the shrinkage that occurs during the polymerization reactions in resin-based restorative materials. This shrinkage occurs when the molecules in the polymer network come closer together, resulting in a reduction in volume compared to the original monomers. 19 As a consequence, this shrinkage generates stress reactions that may have adverse effects on the bond between the cavity walls and the restorative material. If such contraction stress exceeds the bond strength between the RMGIC material and the tooth structure, it can lead to the gap formation between the material and the tooth, which can create potential breaches in the patency of the restoration. These may lead to microbial ingression to the hermetically sealed endodontic chamber/s and compromised treatment outcomes. 19

Ciftçi et al 13 observed higher marginal leakage scores from the hand-mixed composition of Ketac Molar compared to zinc oxide and zinc sulfate-based compound, Cavit-G. One possible explanation for this difference is that despite Cavit-G having lower strength and an extended setting time, it exhibits higher linear expansion coefficients. This property likely led to a more secure and hermetic seal of the cavity, especially when it had sufficient contact time with moisture to properly set. 6

Ketac Molar, which is a metal-free and radio-opaque ionomer material, comes in powder/liquid form. It is available in two formulations: a manual-mixing system or predosed capsules. Ketac Molar exhibits a firm consistency and fluidity, which makes it easy to pack effectively into the cavity. The acidity of the powder contributes to cross-reaction and improved mechanical properties without significantly increasing the initial viscosity of the material. 22

The use of predosed Ketac Molar cement capsules offers several advantages. First, it ensures more consistent mixing ratios, eliminating the potential drawbacks associated with manual mixing. 23 Manual mixing is technique-sensitive and may lead to inconsistencies in material properties. Additionally, manual mixing is time consuming and operator errors in achieving accurate mix ratios can compromise the material's properties and increase the risk of microleakage or marginal degradation. 22

As regards the GIC materials and their variants evaluated in the described reviews, Milani et al 15 observed that Coltosol consistently demonstrated better sealing performance than Compoglass, especially during the early evaluation period. A possible explanation for this could be the nature of the compomers, which are hybrid materials that combine composite resins and GICs. The latter contains bifunctional monomers capable of undergoing both radical polymerizations with methacrylates and an acid-base neutralization reaction with cations released from glass particles facilitated by water. 24 While compomers have higher water sorption capabilities compared to hybrid composites, that mitigates polymerization shrinkage through hygroscopic expansion, 24 they also exhibit significant shrinkage within the first 60 seconds after light curing when the environment is mostly dry 25 which can lead to adhesive failure particularly during the initial curing stage. 25 Moreover, compomers are predominantly hydrophobic, though to a lesser degree than conventional resin composites. 26 They require conventional bonding agents because they do not directly bond to tooth structure like glass ionomers. 26 All of the above factors may play a role in the poor adaptability of the compomers evaluated in comparison to Compoglass. However, there is a need for further research to gain a better understanding of the long-term sealing capabilities and clinical implications of using compomers as temporary restorative materials in endodontic procedures.

## Limitations of the Review

The review has some limitations. First, the studies analyzed primarily relied on dye penetration as a means to assess microleakage. While dye penetration can offer a quantitative measure of microleakage, it does not fully illustrate the complexity of bacterial infiltration that occurs in clinical situations. Several other microleakage assessments, such as the fluid filtration technique quantifies the fluid flow rate through the restoration interface, can provide basic leakage information. Scanning electron microscopy can also identify minute gaps or voids at the interface with high-resolution imagery. 27 Assessment employing electrochemical impedance spectroscopy can also offer a distinct approach by measuring alterations in electrical resistance at the interface, indicative of microleakage presence. 28 Additionally, laser fluorescence, leveraging laser-excited dye, can detect leaks in a nondestructive manner with some limitations. Complementing these techniques is the micro-computed tomography that stands out by generating intricate three-dimensional reconstructions of both the restoration 29 and adjacent tooth structures, thereby offering a comprehensive visualization of microleakage. Each method uniquely contributes to the detailed microleakage assessment, which is crucial for advancing dental restoration practices. Hence, future studies must employ these methods to allow for a thorough microleakage assessment, enhancing restorative material development, leak detection, and predicting restoration longevity. Moreover, methods that more accurately mimic real-life conditions and microbial challenges should be considered in prospective studies.

Second, the use of loading cycles as a measurement in the reviewed studies may not entirely reflect the dynamic forces that restorations are subjected to in actual clinical settings. The mechanical stresses experienced by temporary restorations in the mouth can be diverse and complex, and the loading cycles employed in the studies may not fully replicate these conditions.

## Conclusion

Zinc oxide-based materials, including Cavit, Coltosol, and Caviton, have demonstrated superior coronal sealing effectiveness over certain GIC in controlled settings. Their premixed nature ensures consistent application and hygroscopic properties improve cavity sealing. However, the focus on dye penetration tests for microleakage *in vitro* may not fully represent the risk of bacterial infiltration. Thus, *in vivo* studies are crucial for validating these findings in clinical contexts.
